# An artificial intelligence–enabled Holter algorithm to identify patients with ventricular tachycardia by analysing their electrocardiogram during sinus rhythm

**DOI:** 10.1093/ehjdh/ztae025

**Published:** 2024-04-03

**Authors:** Sheina Gendelman, Eran Zvuloni, Julien Oster, Mahmoud Suleiman, Raphaël Derman, Joachim A Behar

**Affiliations:** Faculty of Biomedical Engineering, Technion-IIT, Julius Silver Building, Haifa 3200003, Israel; Faculty of Biomedical Engineering, Technion-IIT, Julius Silver Building, Haifa 3200003, Israel; IADI, U1254, Inserm, Université de Lorraine, Nancy, France; CIC-IT 1433, Université de Lorraine, Inserm, CHRU de Nancy, Nancy, France; Department of Cardiology, Rambam Medical Center, HaAliya HaShniya St 8, PO Box 9602, Haifa 3109601, Israel; Technion Ruth and Bruce Rappaport Faculty of Medicine, HaAliya HaShniya St 8, PO Box 9602, Haifa 3109601, Israel; Department of Anesthesiology, Rambam Medical Center, HaAliya HaShniya St 8, PO Box 9602, Haifa 3109601, Israel; Faculty of Biomedical Engineering, Technion-IIT, Julius Silver Building, Haifa 3200003, Israel

**Keywords:** Ventricular tachycardia, Sudden cardiac death, Electrophysiology, Holter, Machine learning

## Abstract

**Aims:**

Ventricular tachycardia (VT) is a dangerous cardiac arrhythmia that can lead to sudden cardiac death. Early detection and management of VT is thus of high clinical importance. We hypothesize that it is possible to identify patients with VT during sinus rhythm by leveraging a continuous 24 h Holter electrocardiogram and artificial intelligence.

**Methods and results:**

We analysed a retrospective Holter data set from the Rambam Health Care Campus, Haifa, Israel, which included 1773 Holter recordings from 1570 non-VT patients and 52 recordings from 49 VT patients. Morphological and heart rate variability features were engineered from the raw electrocardiogram signal and fed, together with demographical features, to a data-driven model for the task of classifying a patient as either VT or non-VT. The model obtained an area under the receiving operative curve of 0.76 ± 0.07. Feature importance suggested that the proportion of premature ventricular beats and beat-to-beat interval variability was discriminative of VT, while demographic features were not.

**Conclusion:**

This original study demonstrates the feasibility of VT identification from sinus rhythm in Holter.

## Graphical Abstract

**Graphical Abstract:**
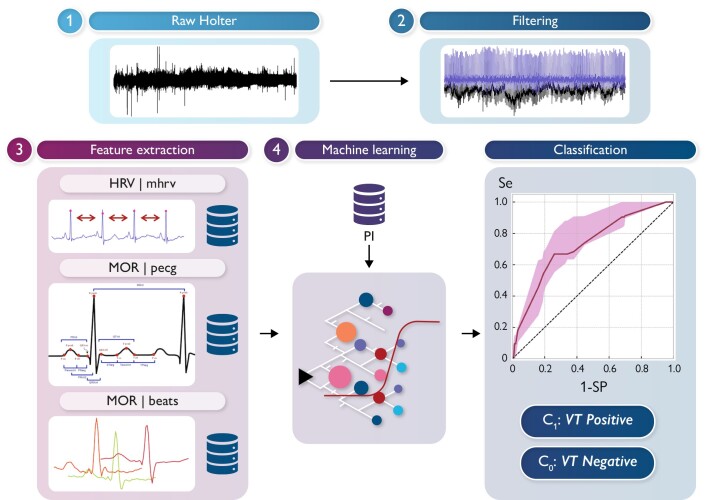

## Introduction

Ventricular tachycardia (VT) is an abnormal rapid heart rhythm originating from the ventricles. The normal heart rate is usually between 60 and 100 b.p.m. In VT, the ventricles beat rapidly, typically between 120 and 300 b.p.m., and are no longer synchronized with the atrium.^1^ Ventricular tachycardia is a serious condition and requires urgent treatment. If left untreated, it can be a major cause of sudden cardiac death.^2^ Ventricular tachycardia may be paroxysmal, which means sudden episodes of abnormal heart rhythms will occur intermittently and spontaneously. This makes identifying individuals with VT a challenge. Researchers have developed machine-learning (ML) algorithms to support the detection of VT and ventricular fibrillation events in long-term continuous electrocardiogram (ECG) recordings such as Holter.^3,4^ While these studies had relatively high performance (although on modest-sized data sets), there is, to our knowledge, no research that has attempted to diagnose VT from non-VT ECG segments. We hypothesize that it is possible to identify patients with VT during sinus rhythm by leveraging a continuous 24 h Holter and artificial intelligence. Related attempts in the context of atrial fibrillation (AF) were recently reported by Attia *et al*.^5^ and Biton *et al*.,^6^ suggesting that artificial intelligence is capable of identifying characteristic changes in the ECG that are associated with the past or future manifestations of an arrhythmia.

## Methods

### Database elaboration

**Figure 1:**
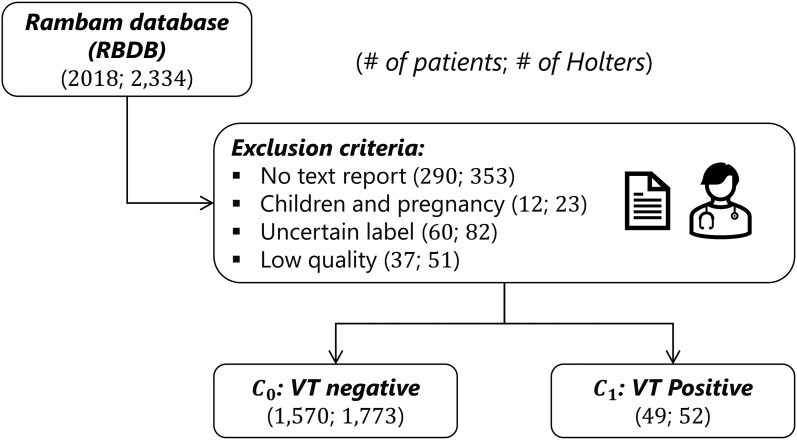
Data set elaboration for the Rambam database. $C_{0}$: non-ventricular tachycardia recordings and $C_{1}$: ventricular tachycardia recordings. Exclusion criteria were applied, reducing the final number of patients and Holter recordings in both classes.

The study protocol was approved by the Rambam ethics committee (RMB-D-0057-23). The Rambam database (RBDB) was obtained from the Rambam Hospital Holter Clinic, Haifa, Israel. The Holter recordings were collected retrospectively from the clinic between August 2017 and November 2021, totalling 2018 patients and 2334 Holter ECG recordings (*Figure 1*). The 24-h Holter ECGs were recorded and analysed using Lifecard CF (Spacelabs Healthcare, WA, USA), which provided a three-lead ECG recording. Pathfinder SL software (Spacelabs Healthcare) was used for data analysis and overread by the local electrophysiologists at the Rambam Cardiology Department. The median and interquartile duration of the recordings was 24.05 h (22.02–25.17 h), the median and interquartile age was 68.0 (51.0–77.0), and 60% of the patients were males. Holter open text reports, i.e. examination reports, including the summary interpretation from the cardiologist, were available for 1981 recordings (84% of the Holters in the database). The exclusion criteria were as follows: absence of open text report, children (<18 years old), pregnancy, and recordings of low quality. Signals were split into 30 min windows, and each window signal quality was measured by using the bsqi.^7^ High-quality windows were defined with a bsqi >0.8. If less than six windows per recording were found to be of high quality, the whole recording was classified as low quality and thus excluded. Supplementary material online, *Figure S1* shows the patient information (PI) of the included data. In order to label the recordings as either VT or non-VT, we first reviewed the text reports of the Holter examinations and retained those flagging VT. These Holters were then manually examined by co-author R.D. to find traces in the ECG that matched the following VT definition: a cardiac arrhythmia with three or more consecutive complexes originating in the ventricles at a rate >100 b.p.m.^8^ Since VT is part of the wide complex tachycardias (WCTs) group, which includes, among others, supraventricular tachycardia with blocks, antidromic tachycardias (Wolff–Parkinson–White syndrome), etc., we needed to differentiate between these WCT types. Although several algorithms have been developed to make the distinction based on 12-lead ECGs, there are no such algorithms for a Holter monitor. Thus, we relied on the following elements to differentiate VT: length of a QRS complex longer than 120 ms, absence of an anterograde P-wave, an axis and duration different from the basic complex, depolarization >40 or 50 ms (or 70 ms, if rS-wave), and presence of a fusion complex.^8–15^ If there was any uncertainty about the nature of the WCT, we concluded that it was not VT. Moreover, there was no distinction between non-sustained VT and sustained VT in these settings. A manual review of Holter served the purpose of labelling the ECG sections in VT. Indeed, since we were interested in this research in identifying VT patients by analysing their ECG during sinus rhythm, we discarded these VT windows in our experiments. Out of the 52 VT-positive recordings based on the text report, we found VT segments in 38 recordings through manual examination and excluded these windows from the analysis. The VT-positive recordings were considered to be the positive class, denoted $C_{1}$. All other recordings constituted the negative class, denoted $C_{0}$.

### Train–test split

Due to the small size of $C_{1}$, a nested cross-fold validation approach was taken. A four-fold cross-validation (inner loop) was performed 10 times (outer loop), each time by rotating the train–test split. For each outer loop split, in $C_{1}$, 10 patients were randomly chosen for the test set and the remaining 39 were used for the training. In $C_{0}$, a total of 130 patients were randomly chosen for the test set and the remaining 1440 were used for the training. There was no information leakage and all recordings of a given patient were included in the training or test set. Each recording was split into windows of 30 min, leading to an average of 46.2 windows per recording. The quality of the ECG was assessed using the bsqi^7^ signal quality index. The bsqi score was computed for each 30 min window. Windows with a bsqi <0.8 were excluded. The RBDB consisted of 84 330 windows, among which 11 071 (13.13%) were excluded because of a low bsqi. Windows belonging to Class $C_{1}$, 64 in total, thus containing annotated VT event(s), were excluded.

### Pre-processing and feature extraction

Pre-filtering of the raw ECG time series was performed to remove the baseline wander and high-frequency noise. Specifically, a zero-phase second-order infinite impulse response bandpass filter with a passband of 0.67–100 Hz was used. A notch filter was used to remove the power-line interference. The filter was set to 50 Hz, which corresponds to the power-line frequency in Europe and Israel. Heart rate variability (HRV) and morphological (MOR) features were extracted for each window using PhysioZoo HRV^16,17^ and PhysioZoo ECG^18^ software. The HRV features are based on the variability of the intervals between each two consecutive heartbeats. A total of 23 HRV features previously implemented in Behar *et al*.^17^ and Chocron *et al*.^16^ were used (see Supplementary material online, *Table S3*). A total of 22 ECG waveform features were engineered (see Supplementary material online, *Table S4*). For each feature, we computed, for a given window, the mean, median, minimum, maximum, and the interquartile range. In addition, beats were classified using the open-source heartbeat classification algorithm by Llamedo and Martinez^19^ as normal, supraventricular, ventricular, fusion, or none (see Supplementary material online, *Table S4*). Beats were classified as none if the classifier was unable to assign a label to the beat. The ratios of each beat type to the total number of beats in a window were added to the MOR feature set, resulting in a total of 115 MOR features. In addition, four PI features available from the patient file were used, namely, age, sex, body mass index (BMI), and smoking status (see Supplementary material online, *Figure S1*).

### Machine learning

Five models that consisted of different feature type combinations were evaluated. A description of the models is provided in Supplementary material online, *Table S5*. An XGBoost^20^ classifier was trained, and hyperparameter tuning was performed using Bayesian search for 50 iterations. During the training, the windows were stratified by patients. The hyperparameters yielding the best area under the receiver operating characteristic curve (AUROC) performance on the validation sets were selected. The minimum redundancy maximum relevance (mRMR) algorithm^21^ was used to rank feature importance and enable feature selection. The energy threshold of mRMR was searched as a hyperparameter. Given the XGBoost probability output per window, each patient was classified as either VT or non-VT by computing the median probability over the entire patient’s windows.

### Statistical analysis and performance measures

Statistical analysis was performed on the features extracted from the RBDB data set. The non-parametric Mann–Whitney rank test was used to determine whether the individual features were significantly different between recordings in Classes $C_{0}$ and $C_{1}$. For all features, a *P*-value cut-off of *P* < 0.05 was used as a significance criterion. The model performance was evaluated using the AUROC score.

## Results

The database RBDB includes 2018 patients and 2334 Holter ECG recordings (*Figure 1*) from patients recorded during routine care. Recordings were annotated as $C_{0}$: non-VT or $C_{1}$: VT recordings. Three types of features were used: HRV, MOR, and PI. A set of five models (*Table 1*) using different feature types were trained within a nested cross-validation framework.

**Table 1:** Test performance for the per recording classification task

Model (feature sets included)	Test AUROC
1 (PI)	0.49 ± 0.07
2 (HRV)	0.74 ± 0.07
3 (MOR)	0.74 ± 0.09
4 (HRV, MOR)	0.76 ± 0.07
5 (PI, HRV, MOR)	0.70 ± 0.05

**Figure 2:**
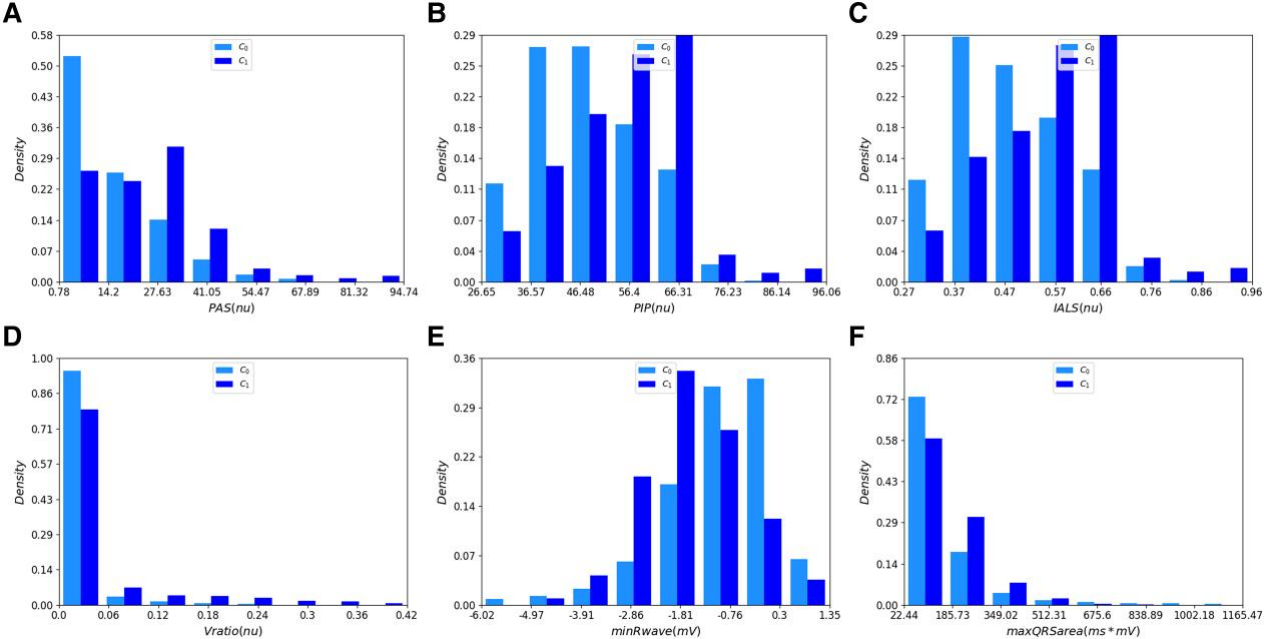
Histograms of the three heart rate variability (*A–C*) and three morphological (*D–F*) features with the lowest *P*-value. The 1st and the 99th percentiles of the data are removed for display purposes. The confirmed ventricular tachycardia recordings are denoted $C_{1}$ and the non-ventricular tachycardia recordings are denoted $C_{0}$.

The Mann–Whitney rank test performed for individual features for $C_{1}$ and $C_{0}\;$ rejected the null hypothesis for 22 out of 23 HRV features and for 106 out of 115 MOR features. Among the HRV features, the *PAS*, *PIP*, and *ILAS* yielded the lowest *P*-values. Among the MOR features, the proportion of premature ventricular contraction (PVC) beats (*Vratio*), minimum R-peak amplitude (*minRwave*), and maximum QRS area ($QRS_{Area}$) yielded the lowest *P*-values. *Figure 2* shows the distributions of these three HRV and three MOR features with the lowest *P*-values. The median and standard deviations for HRV and MOR features are presented in Supplementary material online, *Tables S1* and *S2,* respectively. For the PI features, the null hypothesis was rejected for all four features, namely, age, sex, BMI, and smoking history.

**Figure 3:**
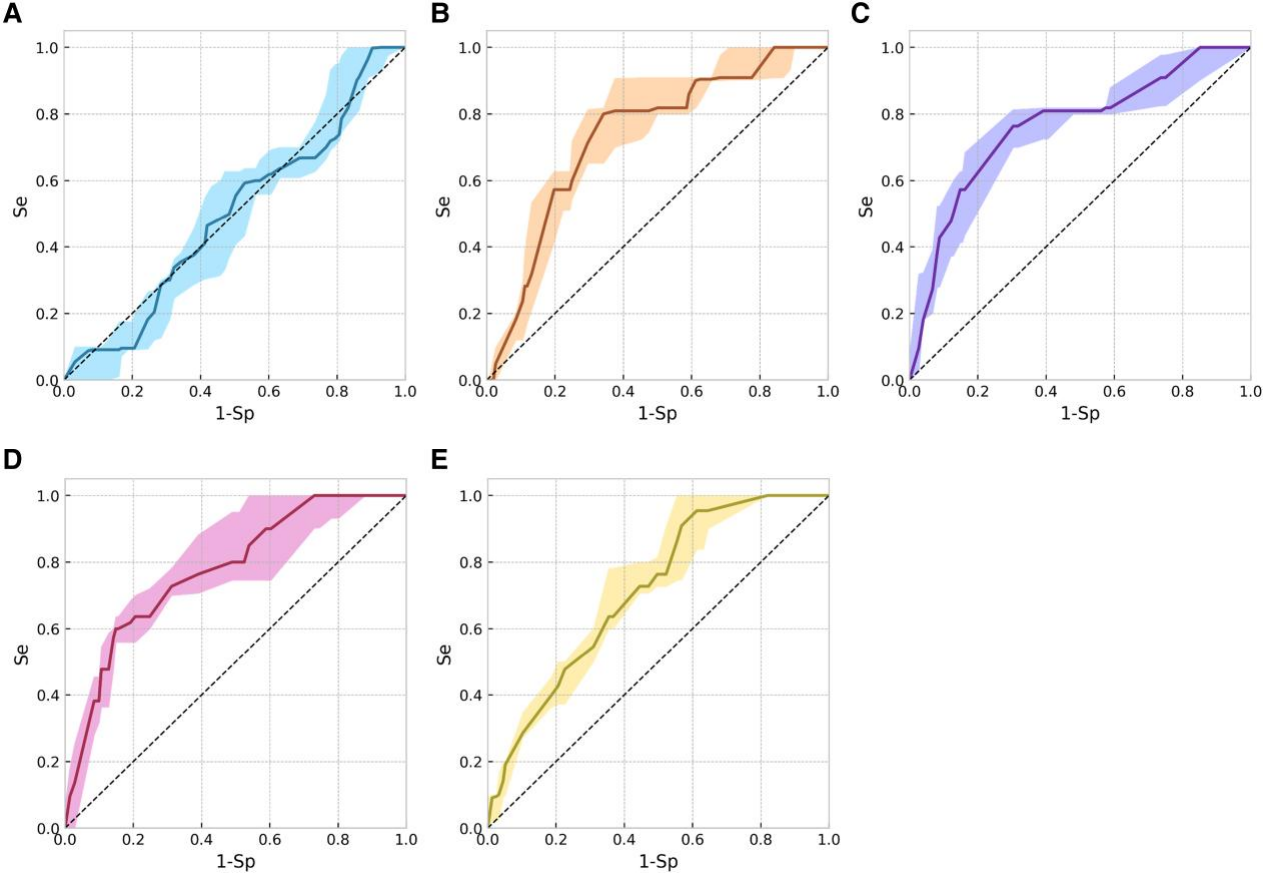
Receiver operating characteristic curves for the per patient classification task and as evaluated on the test sets, with the median (curve) and interquartile range (envelope). The models presented in Panels *A*, *B*, and *C* are trained with one type of feature, i.e. patient information, heart rate variability, or morphological feature, respectively. The model presented in Panel *D* is trained with heart rate variability and morphological features. The model presented in Panel *E* is trained with all feature types, i.e. patient information, heart rate variability, and morphological features.

For the per recording classification task, Model 4, i.e. the combination of HRV and ECG morphology–based model, performed best with an AUROC of 0.76 ± 0.07. *Table 1* summarizes the AUROC on the test sets of RBDB, for each one of the splits. *Figure 3* presents the ROC curves for all the models. A feature importance rating, shown in Supplementary material online, *Figure S2*, reflects that some features particularly stand out. The most predictive MOR features in Model 3 (based solely on MOR features) included *minRwave*, Vratio, and $medianQRS_{int}$. Similarly, for HRV features in Model 2 (based solely on HRV features), the most predictive features included *IALS* and *PAS*, reflecting the degree of sinus rhythm fragmentation.^22^ Consistently, these features remained among the most important predictors in the combined model as well.

## Discussion

In 2019, Attia *et al*.^5^ presented an algorithm for the diagnosis of patients with AF during sinus rhythm from the 12-lead ECG. In 2021, Biton *et al*.^6^ proposed the ML algorithm for the task of AF risk prediction within 5 years from the 12-lead ECG examination. The model combined feature engineering, deep learning, and clinical data. They achieved an AUROC curve of 0.91. Thus, a diagnosis of AF from the non-AF segment as well as AF risk prediction was achieved using 12-lead ECG. In 2022, Singh *et al*.^23^ proposed a deep-learning architecture that was trained to predict a short-term AF from ambulatory monitoring ECG. They used 39 964 Holters with a duration of 7–15 days for training and validation, 9993 as an internal test set, and 5808 as an external test set. The first 24 h from the Holter were used to derive inputs, while the following days were used to assign a label. They achieved an AUROC, Se, and Sp of 79.4, 76, and 69%, respectively, and 75.8, 78, and 58% on the external test set. These recent studies imply structural changes in the ECG that are associated with an existing or impending arrythmia. To our knowledge, no research has attempted such experiments for VT leveraging Holter ECG recordings.

The main contribution of our study is the demonstration of the feasibility of identifying VT from sinus rhythm during prolonged ECG recordings. The best model, utilizing both HRV and MOR features, achieved an AUROC of 0.76. Furthermore, an investigation of the importance of the HRV and MOR features highlighted that PVC-related features were highly predictive. The PVCs were more common in the recordings of VT patients, leading to an increased irregularity of the beat-to-beat interval and major changes in the morphology of the R-wave. The minimum R-peak amplitude was also found to be an important feature and may also be associated with individuals having a high proportion of PVCs. These observations are coherent with individuals with an increased PVC ratio having an association with an increased risk of cardiovascular diseases.^8^ Some pieces of experimental and clinical evidence support the notion that increased sympathetic activity can initiate, or at least facilitate, VT.^24–26^ Furthermore, the degree of fragmentation of the cardiac inter-beat interval time series increases significantly as a function of age in healthy population as well as in patients with some cardiac conditions such as coronary artery disease.^22^ Consequently, the HRV features may capture this increased sympathetic activity and/or the presence of a cardiovascular pathological substrate.

We analysed 50 recordings from non-VT patients that were incorrectly classified with a high probability of VT and thus false positives (FPs). The mean patient age for these recordings was 70.84, and a total of 34 out of the 50 FP recordings had either suspected or known underlying cardiovascular diseases, or a low bsqi along the recording. Specifically, of the 50 recordings, 21 recordings were documented with underlying AF or atrial flutter (AFL), 14 recordings were documented with atrial tachycardia (AT), and 5 recordings had a low bsqi, which led to the exclusion of ≥50% of those recordings’ windows. Additionally, 16 of the recordings were documented with premature atrial contractions (PACs). Furthermore, among those 50 recordings, 32 were documented with an unusually high PVC rate. Since AF, AFL, and AT are characterized by rapid and irregular rhythm, they are more likely to produce WCT in the presence of aberrant intraventricular conduction, and these rhythm patterns may mislead the model decision. We also analysed 10 recordings from VT patients who had the lowest VT probability and thus were false negatives. The mean age of these patients was similar to the mean age of all VT patients, at 66.4 compared with 69.4 years. To further investigate what features were most discriminative, we analysed 10 VT recordings with the highest VT probability, i.e. true positive (TP) recordings, and 50 non-VT recordings with the lowest VT probability, i.e. true negative (TN) recordings. The majority of the TN recordings (46) was not documented with any underlying cardiovascular diseases, and none of them had a low bsqi. Specifically, one patient was suspected to have AT and three patients had AF or AFL. Nevertheless, there were 15 recordings documented with PVCs and 18 recordings with PACs (with 11 cases having both PVCs and PACs). The mean age of the TN patients’ recordings was 45.54 years, while the mean age of the TP patients’ recordings was 77.2 years. By visualizing the examples of the TN and TP recordings, it may be observed that a repeated PVC, a wide QRS, and irregularity in the morphology of the R-wave are characteristic of TP vs. TN. This is coherent with the feature importance reported in Supplementary material online, *Figure S2*.

Our study had limitations. The best model reached AUROC = 0.76, which demonstrates that valuable information is embedded in the ECG time series. This performance is, however, still insufficient for a clinical application, and further research is warranted to improve the model. In addition, the positive VT labels in this study were mainly based on open text Holter reports as available in the hospital electronic medical record system. Such settings were found to be optimal with respect to the available information in our database, yet a less minimal approach would be to select the positive labels according to confirmed recordings consisting of VT events. It may also be limited for individuals who changed medical centres over the years. Besides, we could not distinguish between sustained and non-sustained VT. Another aspect is the electrode placement, which can differ across medical centres. Indeed, contrary to the 12-lead ECG examination, the lead placement of Holters is not standardized. This could lead to limitations in the generalization performance of such a model due to this distribution shift. Patient history, such as information on cardiomyopathies, could be included in the model since these are risk factors. Finally, the deep-learning approaches that we experimented with did not lead to improved performance. This is likely due to the relatively low number of examples in the positive class (52 recordings).

This research demonstrated the feasibility of identifying individuals with VT from sinus rhythms long-term ECG. The feature engineering-based model obtained an AUROC of 0.76 ± 0.07. Feature importance suggested that the proportion of premature ventricular beats and beat-to-beat interval variation variability was discriminative of VT. The demographic features were not discriminative and redundant to the morphological ones. A promising direction to improve the performance of the model would be to gain access to a higher number of VT recordings, which would enable the model to better learn or even enable the development of a deep-learning approach. In addition, in order to reduce the number of FPs, the intended use of the model could be focused on specific groups that are known to be at risk, such as individuals with a history of myocardial infraction or with a low ejection fraction. In this study, we did not have access to these variables. Finally, in line with recent efforts in the research community, the model should be validated on data sets collected at multiple medical centres in order to demonstrate generalizability.^27^ The final model should then be evaluated prospectively within an intended use clinical environment. The resulting improved model could serve as a new artificial intelligence–based approach that may support the better triage of individuals at risk of VT from a standard Holter examination.

## Lead author biography


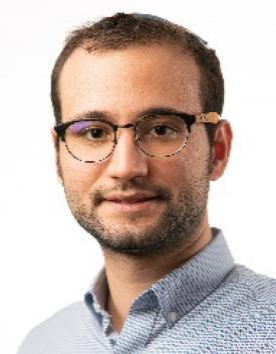


Dr Joachim A. Behar founded the Artificial Intelligence in Medicine Laboratory (AIMLab.) at the Technion Faculty of Biomedical Engineering, Haifa, Israel, in 2019. His work involves the research of medical artificial intelligence to benefit patient care and includes the development of ML algorithms for analysing large medical unstructured data, i.e. physiological time series and medical images, with an emphasis on early diagnosis in the medical disciplines of cardiology, sleep medicine, and ophthalmology. He has made significant contributions to the discovery of diagnostic biomarkers in cardiology and sleep medicine. He serves as an editor for the journal *Physiological Measurement*.

## Supplementary Material

ztae025_Supplementary_Data

## Data Availability

The data that support the findings of this study included raw ECG and clinical variables. Data may be made available for non-commercial academic use from the authors with permission from the Rambam Hospital. Please contact the corresponding author for such requests.
