# Supplementary material for: An artificial intelligence–enabled Holter algorithm to identify patients with ventricular tachycardia by analysing their electrocardiogram during sinus rhythm
Source: Eur Heart J Digit Health. 2024 Apr 3;5(4):409–15. doi: 10.1093/ehjdh/ztae025 (PMC11284005; doi:10.1093/ehjdh/ztae025)
Supplement: ztae025_Supplementary_Data [file ztae025_supplementary_data.pdf]

Supplementary material

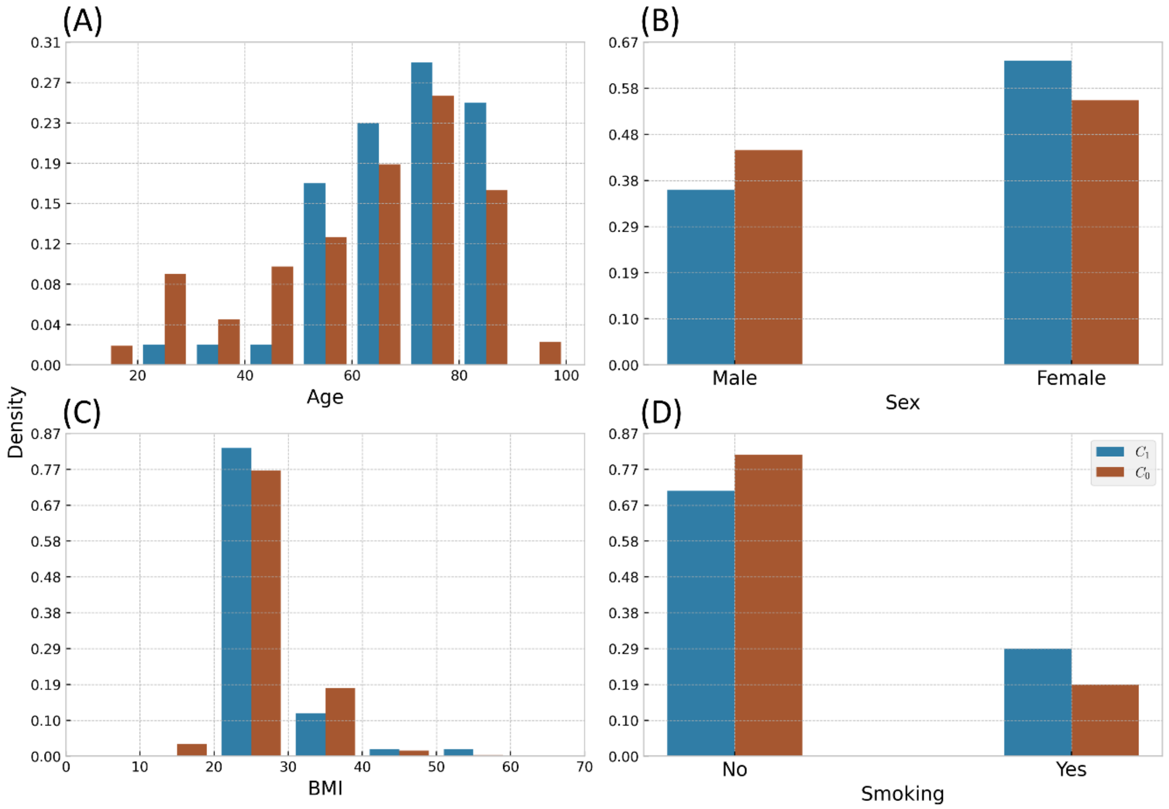

Figure S1. Patient information (PI) feature distributions in the Rambam database (RBDB): A) age, B) sex, C) body mass index (BMI), and D) smoking status. Feature distributions were computed after applying the exclusion criteria. The column colours separate between VT-positive (C_1, blue, n=49) and VT-negative (C_0, orange, n=1,570) patients.

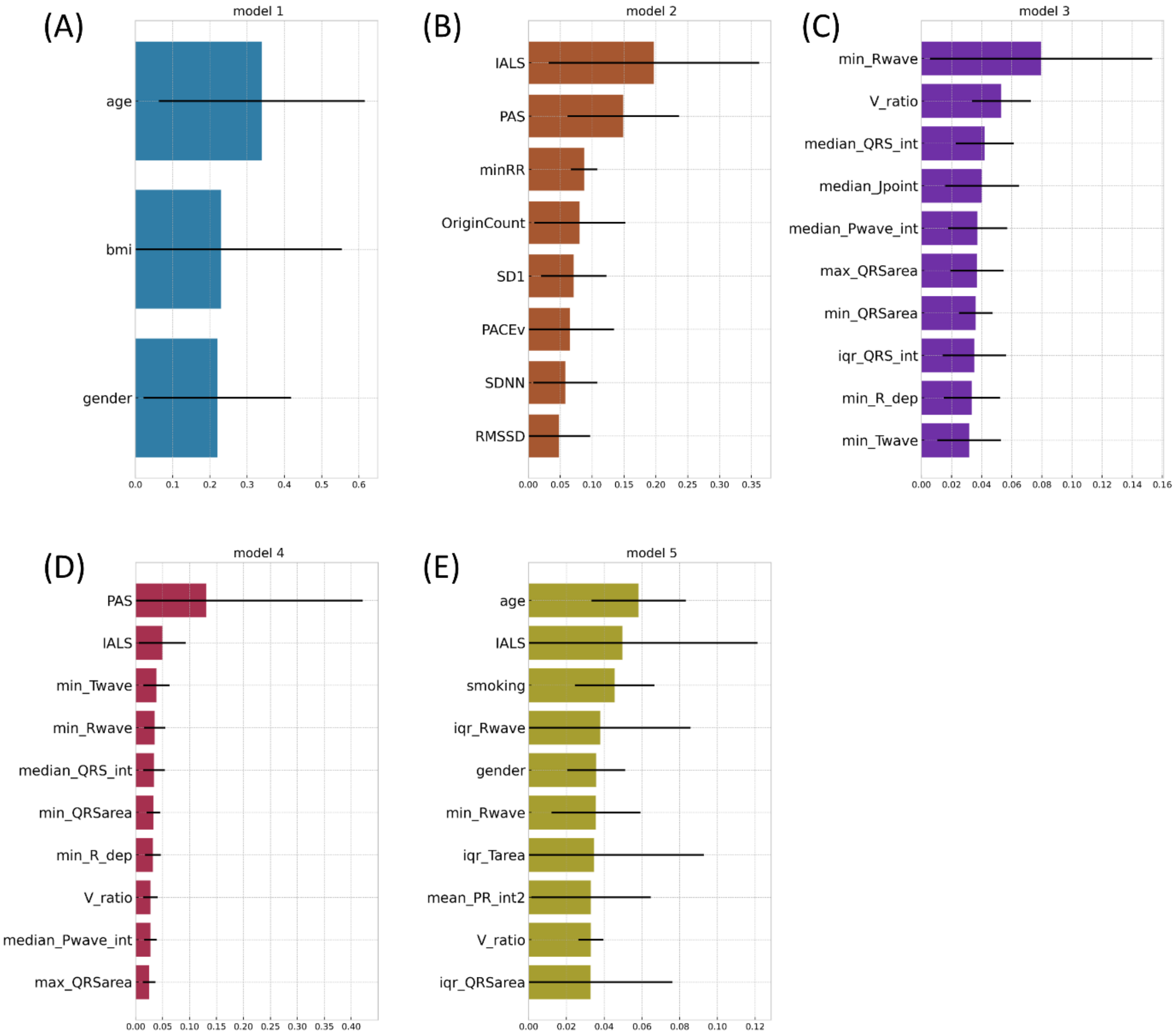

Figure S2. Feature importance for the XGBoost classifier for all models for with the mean and the std over all splits of the outer loop.

Table S1. Summary of the statistical HRV features analysis, for each feature there is a p-value, median and IQR for the positive class (VT) and median and IQR for the negative class (Non-VT).

	P-value	VT	Non-VT
**cosEn**	<0.001	-1.99(0.88)	-2.1(0.54)
**AFEv**	0.01	-901.5(1314.25)	-1032.0(1105.5)
**OriginCount**	<0.001	677.0(1273.5)	976.0(1097.0)
**IrrEv**	<0.001	103.0(198.5)	44.0(84.0)
**PACEv**	<0.001	35.0(219.75)	1.0(40.0)
**AVNN**	<0.001	0.84(0.27)	0.85(0.25)
**minRR**	<0.001	0.37(0.25)	0.41(0.32)
**medHR**	<0.001	71.65(24.79)	70.59(21.58)
**SDNN**	<0.001	0.11(0.11)	0.08(0.08)
**SEM**	<0.001	0.0(0.0)	0.0(0.0)
**PNN20**	<0.001	55.95(57.45)	43.24(45.08)
**PNN50**	<0.001	25.07(60.29)	10.5(31.52)
**RMSSD**	<0.001	0.12(0.19)	0.05(0.09)
**CV**	<0.001	0.13(0.13)	0.09(0.09)
**SD1**	<0.001	0.09(0.13)	0.04(0.07)
**SD2**	0.031	0.11(0.1)	0.1(0.08)
**sq_map_intercept**	<0.001	0.71(0.45)	0.72(0.43)
**sq_map_linear**	<0.001	-1.68(0.54)	-1.69(0.5)
**sq_map_quadratic**	1	1.0(0.0)	1.0(0.0)
**PIP**	<0.001	56.94(17.82)	47.26(16.75)
**IALS**	<0.001	0.57(0.18)	0.47(0.17)
**PSS**	<0.001	59.57(28.63)	47.69(28.27)
**PAS**	<0.001	24.58(21.81)	11.8(16.15)

Table S2. Summary of the statistical MOR features analysis, for each feature there is a p-value, median and IQR for the positive class (VT) and median and IQR for the negative class (Non-VT).

	p-value	VT	Non-VT
**mean_Pwave_int**	<0.001	114.3(22.75)	110.99(21.63)
**median_Pwave_int**	<0.001	110.0(20.0)	105.0(20.0)
**min_Pwave_int**	<0.001	50.0(15.0)	55.0(20.0)
**max_Pwave_int**	<0.001	285.0(25.0)	285.0(25.0)
**iqr_Pwave_int**	<0.001	20.0(40.0)	15.0(35.0)
**mean_PR_int**	<0.001	194.22(58.32)	181.97(40.08)
**median_PR_int**	<0.001	185.0(60.0)	175.0(40.0)
**min_PR_int**	<0.001	105.0(25.0)	110.0(20.0)
**max_PR_int**	<0.001	335.0(0.0)	335.0(0.0)
**iqr_PR_int**	<0.001	30.0(55.0)	25.0(45.0)
**mean_PR_seg**	<0.001	78.63(35.86)	69.2(27.18)
**median_PR_seg**	<0.001	75.0(35.0)	65.0(35.0)
**min_PR_seg**	<0.001	45.0(0.0)	45.0(0.0)
**max_PR_seg**	<0.001	245.0(80.0)	230.0(95.0)
**iqr_PR_seg**	<0.001	20.0(40.0)	15.0(20.0)
**mean_PR_int2**	<0.001	179.18(63.12)	162.84(44.01)
**median_PR_int2**	<0.001	175.0(70.0)	155.0(45.0)
**min_PR_int2**	<0.001	85.0(25.0)	90.0(30.0)
**max_PR_int2**	<0.001	365.0(60.0)	355.0(60.0)
**iqr_PR_int2**	<0.001	28.75(65.0)	15.0(55.0)
**mean_QRS_int**	0.01	95.37(38.41)	92.74(24.72)
**median_QRS_int**	0.727	90.0(45.0)	90.0(25.0)
**min_QRS_int**	<0.001	45.0(20.0)	50.0(20.0)
**max_QRS_int**	<0.001	265.0(120.0)	240.0(130.0)
**iqr_QRS_int**	<0.001	15.0(20.0)	15.0(15.0)

**mean_QT_int**	<0.001	408.73(63.73)	402.97(62.4)
**median_QT_int**	0.006	405.0(65.0)	400.0(65.0)
**min_QT_int**	<0.001	230.0(80.0)	250.0(105.0)
**max_QT_int**	<0.001	630.0(125.0)	605.0(140.0)
**iqr_QT_int**	<0.001	30.0(40.0)	20.0(25.0)
**mean_Twave_int**	0.595	198.88(44.53)	197.67(38.7)
**median_Twave_int**	0.71	200.0(50.0)	200.0(45.0)
**min_Twave_int**	<0.001	70.0(30.0)	80.0(40.0)
**max_Twave_int**	<0.001	355.0(80.0)	345.0(85.0)
**iqr_Twave_int**	<0.001	40.0(40.0)	25.0(35.0)
**mean_TP_seg**	<0.001	241.54(221.15)	255.74(207.35)
**median_TP_seg**	<0.001	245.0(235.0)	260.0(225.0)
**min_TP_seg**	<0.001	5.0(0.0)	5.0(0.0)
**max_TP_seg**	<0.001	705.0(460.0)	625.0(375.0)
**iqr_TP_seg**	0.075	95.0(115.0)	95.0(85.0)
**mean_RR_int**	<0.001	841.55(270.04)	846.83(249.72)
**median_RR_int**	<0.001	837.5(280.0)	850.0(260.0)
**min_RR_int**	<0.001	372.5(250.0)	405.0(325.0)
**max_RR_int**	<0.001	1305.0(530.0)	1205.0(420.0)
**iqr_RR_int**	0.003	85.0(145.0)	80.0(85.0)
**mean_R_dep**	<0.001	40.18(16.69)	38.9(13.36)
**median_R_dep**	0.917	35.0(15.0)	35.0(15.0)
**min_R_dep**	<0.001	10.0(5.0)	10.0(5.0)
**max_R_dep**	<0.001	120.0(55.0)	120.0(60.0)
**iqr_R_dep**	<0.001	10.0(15.0)	10.0(10.0)
**mean_QTc_b**	<0.001	448.83(45.21)	437.97(47.44)
**median_QTc_b**	<0.001	446.12(45.22)	435.8(45.85)
**min_QTc_b**	<0.001	266.52(83.3)	279.7(101.71)
**max_QTc_b**	<0.001	679.9(111.72)	655.27(132.8)

**iqr_QTc_b**	<0.001	34.64(50.89)	23.68(32.5)
**mean_QTc_frid**	<0.001	434.65(44.29)	424.32(43.3)
**median_QTc_frid**	<0.001	432.7(44.41)	422.31(42.24)
**min_QTc_frid**	<0.001	253.15(79.34)	269.87(101.68)
**max_QTc_frid**	<0.001	664.47(105.5)	637.88(133.18)
**iqr_QTc_frid**	<0.001	33.24(48.3)	22.7(30.65)
**mean_QTc_fra**	<0.001	433.02(43.56)	423.0(41.62)
**median_QTc_fra**	<0.001	430.77(43.0)	421.16(40.39)
**min_QTc_fra**	<0.001	267.37(74.85)	279.26(92.32)
**max_QTc_fra**	<0.001	651.17(106.97)	625.36(130.01)
**iqr_QTc_fra**	<0.001	30.0(40.0)	20.0(25.0)
**mean_QTc_hod**	<0.001	436.26(37.33)	424.88(40.77)
**median_QTc_hod**	<0.001	433.36(38.31)	422.91(40.03)
**min_QTc_hod**	<0.001	271.47(84.11)	281.77(94.26)
**max_QTc_hod**	<0.001	652.09(103.99)	626.08(128.08)
**iqr_QTc_hod**	<0.001	30.0(40.0)	20.0(25.0)
**mean_Pwave**	<0.001	0.08(0.07)	0.09(0.07)
**median_Pwave**	<0.001	0.08(0.08)	0.09(0.08)
**min_Pwave**	<0.001	0.0(0.0)	0.0(0.0)
**max_Pwave**	0.026	0.39(0.43)	0.38(0.41)
**iqr_Pwave**	<0.001	0.03(0.03)	0.03(0.03)
**mean_Twave**	0.023	0.19(0.19)	0.2(0.2)
**median_Twave**	<0.001	0.16(0.19)	0.19(0.21)
**min_Twave**	<0.001	0.0(0.01)	0.0(0.03)
**max_Twave**	<0.001	1.05(0.81)	0.79(0.76)
**iqr_Twave**	<0.001	0.07(0.07)	0.06(0.05)
**mean_Rwave**	<0.001	0.33(1.18)	0.63(0.87)
**median_Rwave**	<0.001	0.35(1.31)	0.64(0.9)
**min_Rwave**	<0.001	-1.62(1.41)	-0.74(1.38)

**max_Rwave**	0.001	1.78(1.38)	1.71(1.28)
**iqr_Rwave**	<0.001	0.24(0.26)	0.26(0.29)
**mean_STamp**	<0.001	0.07(0.06)	0.06(0.05)
**median_STamp**	<0.001	0.05(0.07)	0.05(0.05)
**min_STamp**	0.071	0.0(0.0)	0.0(0.0)
**max_STamp**	<0.001	0.56(0.46)	0.48(0.45)
**iqr_STamp**	<0.001	0.05(0.06)	0.04(0.05)
**mean_Parea**	0.627	5.19(3.8)	5.09(3.6)
**median_Parea**	0.331	4.94(3.81)	4.76(3.64)
**min_Parea**	<0.001	0.33(0.56)	0.62(1.05)
**max_Parea**	0.001	24.44(24.28)	22.84(23.01)
**iqr_Parea**	<0.001	1.66(1.82)	1.51(1.44)
**mean_Tarea**	<0.001	17.48(19.42)	18.67(18.58)
**median_Tarea**	<0.001	14.57(19.08)	18.1(19.35)
**min_Tarea**	<0.001	1.51(2.5)	2.4(4.15)
**max_Tarea**	<0.001	94.03(84.46)	64.74(62.68)
**iqr_Tarea**	<0.001	5.72(6.46)	5.1(4.7)
**mean_QRSarea**	<0.001	39.17(36.57)	35.47(25.12)
**median_QRSarea**	0.018	37.0(28.84)	34.25(24.59)
**min_QRSarea**	<0.001	10.83(14.76)	17.99(20.63)
**max_QRSarea**	<0.001	148.95(123.68)	100.51(115.9)
**iqr_QRSarea**	<0.001	5.36(5.63)	4.23(3.92)
**mean_Jpoint**	<0.001	-0.06(0.09)	-0.07(0.07)
**median_Jpoint**	<0.001	-0.06(0.09)	-0.07(0.07)
**min_Jpoint**	<0.001	-0.43(0.65)	-0.39(0.7)
**max_Jpoint**	<0.001	0.44(0.69)	0.26(0.74)
**iqr_Jpoint**	<0.001	0.03(0.03)	0.02(0.02)
**N_ratio**	<0.001	0.96(0.19)	0.99(0.08)
**S_ratio**	0.086	0.01(0.05)	0.01(0.05)

**V_ratio**	<0.001	0.01(0.04)	0.0(0.0)
**F_ratio**	<0.001	0.0(0.0)	0.0(0.0)
**U_ratio**	<0.001	0.0(0.0)	0.0(0.0)

Table S3. HRV features and definitions. This table was adapted from Chocron et al(13) and Behar et al(14).

#	**Biomarkers**	**Definition**
1	$CosEn$	Coefficient of sample entropy.(19)
2-5	$AFEv, IrrEV,$ $PACEv, OriginCount$	Measures derived from Lorenz plot to assess irregularities in the RR intervals.
6-7	$SD1, SD2$	The standard deviation of two principal axis of the ellipse on the Poincare plot. (20)
8	$minRR$	The minimal RR interval in the segment.
9	$midHR$	The median heart rate in the segment.
10	$AVNN$	The mean RR interval over the segment.
11	$SDNN$	The standard deviation of the RR intervals over a segment.
12	SEM	Standard error of the mean
13-14	$PNN20, PNN50$	The percentage of RR intervals shorter than 20 and 50 [ms], respectively. (21)
15	$PMSSD$	The root mean square of the successive differences.
16	CV	Coefficient of variation
17-20	$PIP, IALS, PSS, PAS$	Fragmentation measures(7)
21-23	$sq_{map}intercept,$ $sq_{map}linear,$ $sq_{map}quadratic$	Extended parabolic phase space mapping features(22)

Table S4. Morphological ECG biomarkers and their definition.

#	***Biomarkers***	**Definition**
Intervals and Segments		
1	$Pwave_{int}$	Time interval between P-peak and P-offset
2	PR_{int}	Time interval between P-onset and Q-onset
3	$PR2_{int}$	Time interval as defined by Mao at el(23).
4	PR_{seg}	Time interval between P-offset and Q-onset
5	QRS_{int}	Time interval between Q-onset and S-offset
6	QT_{int}	Time interval between Q-onset and T-offset
7	QT_{cB}	Corrected QT interval (QTc) by Bazett(24)
8	QT_{cFrid}	QTc by Fridericia(25)
9	QT_{cF}	QTc by Fragmingham(26)
10	QT_{cH}	QTc by Hodges(27)
11	$Twave_{int}$	Time interval between T-onset and T-offset
12	TP_{seg}	Time interval between T-onset and P-onset
13	RR_{int}	Time interval between consecutive R peaks
14	R_{dep}	Time interval between Q-onset and R-peak
Waves characteristics		
15	$Pwave$	Amplitude difference between R-peak and P-offset
16	$Twave$	Amplitude difference between T-peak and T-offset
17	$Rwave$	R peak amplitude
18	$Pwave_{Area}$	P wave interval area defined as integral between P-onset and P-offset
19	$Twave_{Area}$	T wave interval area defined as integral between T-onset and T-offset
20	QRS_{Area}	QRS interval area defined as integral between Q-onset and S-offset
21	ST_{seg}	Amplitude difference between S-offset and T-onset
22	J_{point}	Amplitude 40ms after S-offset as defined by Hollander et al(28).
Heartbeat classification		
23-27	N-, S-, V-, F-, U-ratio	Ratio from the total number of beats classified, according to Llamedo at el(16).

Table S5. The definition of the five benchmarked ML models. The median and IQR # of the features selected are presented for all the splits.

	PI	HRV	MOR	#features	#significant features (p<0.05)	#selected features (by mRMR algorithm)
						Median [IQR]
**1**	#			4	4	3 [2.25,3]
**2**		#		23	22	9.5 [8.25,10]
**3**			#	115	106	30.5 [29.25,34.75]
**4**		#	#	138	128	35 [32.25,37.75]
**5**	#	#	#	144	132	35.5 [34.25,38.75]
